# Immunogenic cell death and bystander killing: expanding the therapeutic potential of modern antibody-drug conjugates

**DOI:** 10.1080/2162402X.2025.2533488

**Published:** 2025-07-15

**Authors:** Oliver Kepp, Guido Kroemer

**Affiliations:** a Université Paris Cité, Sorbonne Université, Inserm, Centre de Recherche des Cordeliers, Equipe labellisée parla Ligue contre le cancer, Institut Universitaire de France, Paris, France; b Université Paris-Saclay, INSERM US23/CNRS UAR 3655, Metabolomics and Cell Biology Platforms, Institut Gustave Roussy, Villejuif, France; c Institut du Cancer Paris CARPEM, Department of Biology, Hôpital Européen Georges Pompidou, AP-HP, Paris, France

**Keywords:** Cancer, CD47, cell stress, immunotherapy

## Abstract

HER3-DXd and T-DXd are antibody-drug conjugates (ADCs) that induce immunogenic cell death and bystander killing, enhancing antitumor immunity beyond target-specific cytotoxicity. These findings highlight novel mechanisms that can overcome antigen heterogeneity and suggest opportunities for improving ADC design and therapeutic synergy through informed combination with immunotherapies.

Over the past decades, the oncological armamentarium has been substantially expanded, ranging from new cytotoxic chemotherapies to targeted agents. Moreover, in recent years, several modalities of immunotherapy have been integrated into clinical routine, improving responses in a subset of patients with immunologically susceptible tumors. Antibody-drug conjugates (ADCs) represent a chimeric strategy that integrates the specificity of monoclonal antibodies with the potency of cytotoxic payloads to selectively target and eliminate tumor cells. Regardless of the therapeutic approach, it is now widely accepted that the efficacy of anticancer agents relies not solely on direct tumor-killing but also on the ability to engage and activate the immune system. Immunogenic cell death (ICD) is a form of regulated cell death characterized by the release of damage-associated molecular patterns (DAMPs) including calreticulin, ATP, and HMGB1 which serve as potent immunostimulatory cues by acting on pattern recognition receptors expressed on antigen-presenting cells. These signals promote the recruitment and activation of dendritic cells in particular, ultimately priming a cytotoxic T cell-mediated immune response against residual tumor cells. While ICD was initially associated with certain chemotherapeutics (such as anthracyclines, oxaliplatin and taxanes), it has recently emerged as a desirable feature of ADCs, broadening their mechanism of action beyond simple targeted cytotoxicity. Examples of ICD-inducing ADCs include BB-1701, a HER2-antibody conjugated to eribulin^1^; trastuzumab conjugated to emtansine (T-DM1)^2^; brentuximab vedotin equipped with auristatin E (MMAE), a highly potent microtubule-disrupting agent^3^; belantamab mafodotin, a mAb specific for B-cell maturation antigen (BCMA) conjugated with monomethyl auristatin F^4^; D3-GPC2-PBD, which contains a pyrrolobenzodiazepine (PBD) dimer and targets glypican 2 (GPC2) expressed by neuroblastoma^5^; and an antibody targeting TWEAKR conjugated to a kinesin spindle protein inhibitor (KSPi).^6^

Recent studies, including our own,^7,8^ have provided compelling evidence that patritumab deruxtecan (HER3-DXd) and trastuzumab deruxtecan (T-DXd), which are two ADCs targeting HER3 and HER2, respectively, also exert at least part of their antitumor efficacy through the induction of ICD. Both HER3-DXd and T-DXd are monoclonal antibodies linked to the membrane-permeable topoisomerase I inhibitor DXd via a cleavable peptide linker that is effectively processed by cathepsins present within the lysosomal compartment.^9^ Intriguingly, both studies^7,8^ report a robust bystander effect, meaning that not only cells that express the nominal antigen are killed by HER3-DXd and T-DXd, but that also adjacent cells lacking HER3 or HER2 succumb to DXd that is locally released from HER3 or HER2 cells due to DXd’s membrane permeability.

Tsao et al.^8^ demonstrated that the proteolytic activity of cathepsin L (CTSL) allows for the cleavage of the linker between the anti-HER2 antibody and DXd, hence releasing the payload in the extracellular tumor microenvironment even without antigen internalization, thereby overcoming traditional constraints on ADC efficacy in antigen-heterogeneous tumors.^8^ Similarly, our work^7^ documented that HER3-DXd can induce cell death in HER3-negative tumor cells co-cultured with HER3-positive counterparts, correlating with the appearance of free, unbound DXd in the supernatant of such cocultures, as determined by mass spectrometry.

Altogether this diffusion-based bystander killing represents a major benefit as it addresses the Achilles’ heel of conventional ADCs represented by antigen heterogeneity (due to clonal of tumor antigens) that commonly develops when cancer progress under therapeutic pressure. Although bystander effects in ADCs have been previously recognized, the concurrent demonstration of bystander killing and immunogenic cell death was only recently established.^7,8^ Of note the bystander activity can depend on multiple factors, including linker stability, the cell death mechanism induced by the payload, and the enzymatic landscape of the tumor microenvironment. The pronounced bystander effect observed with HER3-DXd and T-DXd likely reflects the membrane permeability of the DXd payload, coupled with efficient lysosomal processing, which together enable cytotoxicity in neighboring tumor cells irrespective of target expression. While similar mechanisms have been described for other ADCs, the extent and consistency of these effects can vary depending on the payload class and tumor context.^9^ Moreover, potential risks of bystander activity, including off-target toxicity and damage to normal cells adjacent to tumor sites, warrant careful consideration during ADC design and clinical development. More importantly, the spillover of DXd-mediated cytotoxicity expands the pool of dying tumor cells and, consequently, the repertoire of antigens to which the immune system may respond (Figure 1), hence fostering a broader and durable antitumor immune memory.

**Figure 1.**
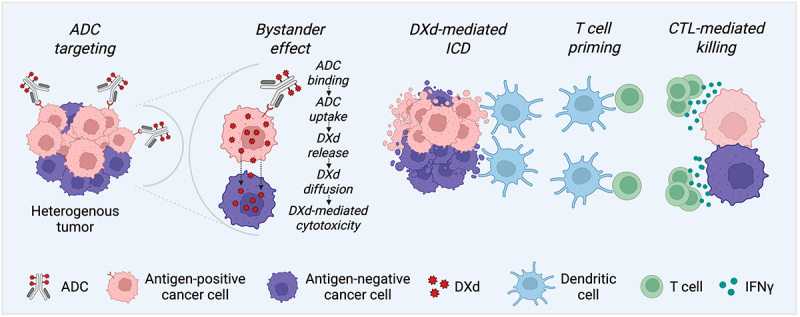
Mechanisms of action of deruxtecan-based ADCs. antibody-drug conjugates (ADCs) such as patritumab deruxtecan (HER3-DXd) and trastuzumab deruxtecan (T-DXd) selectively bind to tumor cells expressing nominal antigen within a heterogeneous tumor microenvironment. Following antigen binding and ADC internalization, the membrane-permeable DXd payload is liberated due to lysosomal processing. Alternatively, extracellular cathepsin activity can cleave the linker, enabling DXd release directly into the tumor milieu. The released payload induces direct tumor cell killing and also diffuses into neighboring cells (regardless of their antigenic makeup), mediating a potent bystander effect. DXd-induced immunogenic cell death (ICD) triggers the release of DAMPs, promoting dendritic cell (DC) maturation, antigen uptake, and cross-presentation. This cascade primes cytotoxic T lymphocytes (CTLs), enabling systemic immune activation and interferon gamma (IFNγ-mediated tumor lysis. The synergy between localized cytotoxicity and immune engagement is a defining feature of the therapeutic efficacy of DXd-coupled ADCs.

Mechanistically, T-DXd was shown to activate immune sensors including STING and TLR4 downstream of ICD-associated DAMP emission, further activating myeloid cells, promoting dendritic cell maturation and priming CD8^+^ T cells.^8^ We observed that, in mice, injections of DXd-conditioned cancer cells conferred protective immunity against tumor rechallenge, indicating the capacity of DXd-treated tumor cells to serve as therapeutic cancer vaccines.^7^ Accordingly, T-DXd reduced the growth of HER2 positive breast cancer in immunocompetent mice and increased the tumor infiltration by cytotoxic T lymphocytes. The anticancer effects of T-DXd were further improved when combined with antibodies blocking CD47 (a ‘don’t eat me’ signal upregulated in response to DXd), collectively enhancing immune-mediated tumor eradication and the establishment of long-term immune memory.^8^

In sum, these findings exemplify the therapeutic potential of modern ADCs such as HER3-DXd and T-DXd, which combine targeted cytotoxicity with robust immunostimulatory effects through ICD induction and bystander killing. We surmise that, as the field advances, it will become increasingly important to optimize ADC payloads not only for specificity and target cell killing, but also for immunogenicity. Bystander killing will be essential. In parallel, strategies combining ADCs with immune checkpoint inhibitors that (re-)invigorate T cell responses or increase the phagocytic potential of ADCs, holds significant promise for eliciting full-blown antitumor immunity, as reflected in a growing number of ongoing clinical trials.^10^ Together, these insights point to a future in which ADCs evolve from precision-guided cytotoxins into fully integrated immunotherapeutic platforms.

## Data Availability

Data sharing is not applicable to this article as no new data were created or analyzed in this study.
